# Fatherhood and Cardiovascular Health, Disease, and Mortality: Associations From the Multi-Ethnic Study of Atherosclerosis

**DOI:** 10.1016/j.focus.2024.100231

**Published:** 2024-05-06

**Authors:** John James F. Parker, Craig F. Garfield, Clarissa D. Simon, Laura A. Colangelo, Michael P. Bancks, Norrina B. Allen

**Affiliations:** 1 Department of Pediatrics, Feinberg School of Medicine, Northwestern University, Chicago, Illinois; 2 Department of Medicine, Feinberg School of Medicine, Northwestern University, Chicago, Illinois; 3 Family and Child Health Innovations Program, Smith Child Health Outcomes, Research and Evaluation Center, Ann & Robert H. Lurie Children's Hospital of Chicago, Chicago, Illinois; 4 Department of Preventive Medicine, Feinberg School of Medicine, Northwestern University, Chicago, Illinois; 5 Department of Epidemiology and Prevention, Wake Forest University School of Medicine, Winston-Salem, North Carolina

**Keywords:** Cardiovascular health, cardiovascular disease, fatherhood, men's health, social influencers of health

## Abstract

**Introduction:**

Emerging literature links fatherhood to men's health but lacks comprehensive assessment of health outcomes, especially among multiethnic populations. This study's objective was to evaluate the associations of fatherhood (age at onset and status) with cardiovascular health scores, incident cardiovascular disease, cardiovascular disease death, and all-cause mortality, examining differences by race/ethnicity.

**Methods:**

The study sample included men from Multi-Ethnic Study of Atherosclerosis, prospective cohort study that enrolled adults aged 45–84 years without known cardiovascular disease at baseline. Cardiovascular health was defined using the American Heart Association's Life's Essential 8 scores (0–100), excluding sleep (cardiovascular health score).

**Results:**

In this sample of 2,814 men, mean age at cardiovascular health assessment was 62.2 years, 82% were fathers, 24% self-identified as Black, 13% self-identified Chinese, 22% self-identified Hispanic, and 41% self-identified White. Fathers who were aged <20 years and 20–24 years at their oldest child's birth had worse overall cardiovascular health than fathers who were aged >35 years (adjusted mean score of 61.1 vs 64.7 [*p*=0.01] and 61.0 vs 64.7 [*p*<0.001], respectively). Fathers had worse overall cardiovascular health (adjusted mean score of 63.2 vs 64.7, *p*=0.03) and more nicotine exposure (63.1 vs 66.6, *p*=0.04) than nonfathers. In age-adjusted models, fathers overall (hazard ratio=0.82; 95% CI=0.69, 0.98) and Black fathers (hazard ratio=0.73; 95% CI=0.53, 0.999) had a lower rate of all-cause mortality rate than nonfathers, but these associations were no longer significant in fully adjusted models.

**Conclusions:**

Fatherhood is a social determinant of health, and understanding its influence may provide opportunities to improve men's health, particularly among men of color.

## HIGHLIGHTS

• Cardiovascular health in older age was worse for fathers than for nonfathers.
• Cardiovascular health was worse for men who became fathers at a young age (i.e., <25 years).
• Fatherhood's association with cardiovascular health and mortality differed by race.
• Black fathers had lower age-adjusted mortality rates than Black nonfathers.
• Incident cardiovascular disease did not differ between fathers and nonfathers.

## Graphical abstract

**Figure:**
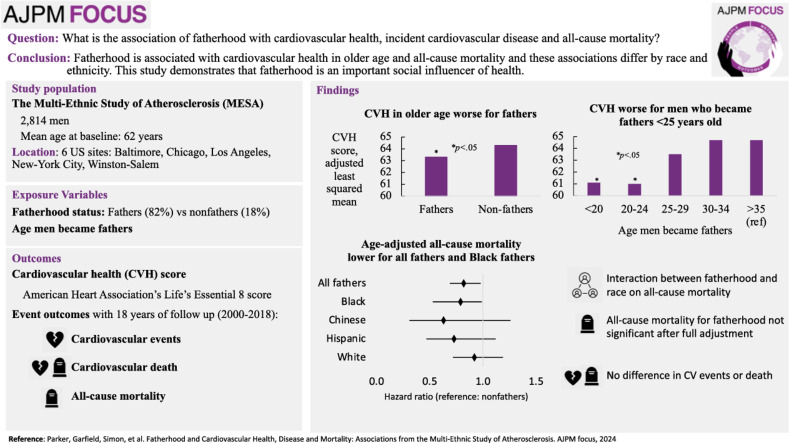
Graphical Abstract for "Fatherhood and Cardiovascular Health, Disease and Mortality: Associations from the Multi-Ethnic Study of Atherosclerosis" by Parker et al.

## INTRODUCTION

In 2021, the life expectancy for men in the U.S. was on average 6 years less than the life expectancy for women, and this discrepancy is most significant for racial and ethnic minorities.^1^ Cardiovascular disease (CVD) is the leading cause of death among men,^2^ and men have worse cardiovascular health (CVH) than women.^3^ CVH is a global summary of health factors and behaviors that have been proven to influence the development of CVD and is measured by the American Heart Association's Life's Essential 8 (LE8).^4^ Men's CVH declines most rapidly from late adolescence through their 30s,^5^ and during this time, most men in the U.S. become fathers.^6^ Notably, fatherhood is increasingly recognized as an influencer of health^7^ and a key experience in the life course of men.^8^

The transition to fatherhood is associated with both positive and negative changes in CVH metrics, such as a decrease in smoking rates,^9^ an increase in BMI,^10^^,^^11^ an increase in fat intake,^12^ and a decrease in physical activity.^9^ In addition, becoming a father at a younger age is associated with CVH metrics^13^ and all-cause mortality.^14^^,^^15^ Being a father has been associated with higher rates of CVD in Chinese men^16^ and lower CVD in cohorts of primarily White participants, suggesting potential differences by race and culture.^17^^,^^18^ Prior studies have found that fatherhood is also associated with lower risk of all-cause mortality^17^^,^^18^ and multiple other health-related factors, such as depression,^19^ marriage,^20^ male infertility,^21^ testosterone level,^22^ and occupation and income,^6^ which highlights the complex relationship between fatherhood and health.

Previous studies that evaluated fatherhood, CVH, CVD, and mortality have not included racially and ethnically diverse populations and lacked comprehensive CVH evaluation.^17^^,^^18^^,^^21^^,^^23^ The Multi-Ethnic Study of Atherosclerosis (MESA) is a unique scientific resource that has collected diverse participant population data on fatherhood and the information needed to calculate LE8. In addition, MESA's longitudinal study has relatively high retention and a large number of recorded disease events.^24^ This study's objectives were to measure the associations between fatherhood onset and status with (1) CVH scores measured by the LE8 framework and (2) incident CVD and all-cause mortality and assess for differences among race and ethnicity using MESA's diverse sample. The authors hypothesized that the association between fatherhood and CVH, CVD, and mortality would differ by race and ethnicity.

## METHODS

### Study Sample

**Figure 1:**
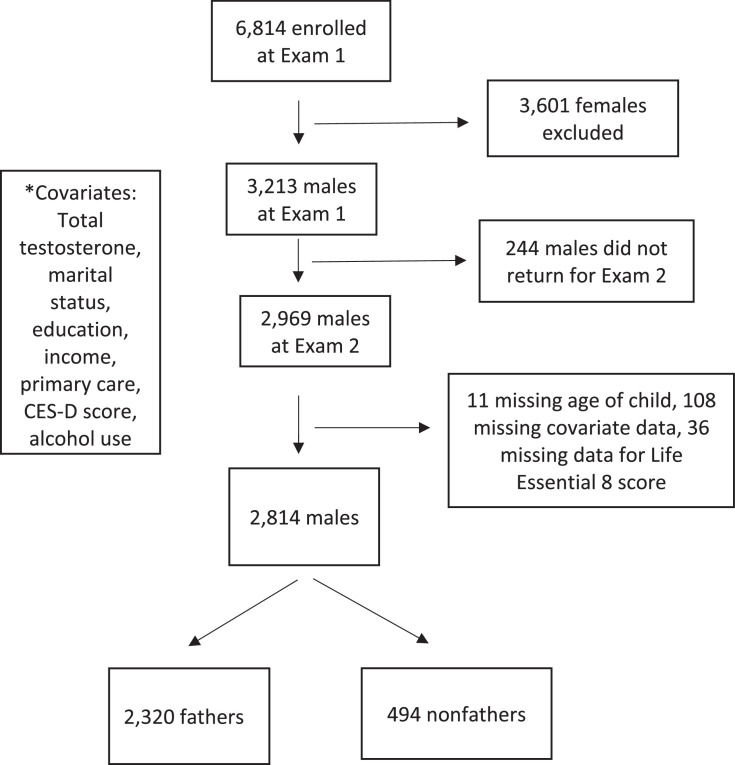
Flow diagram for determining sample size.

MESA is a prospective cohort study of 6,814 persons (47.2% men) aged 45–84 years without known CVD at baseline.^25^ MESA was designed to evaluate subclinical CVD in a diverse sample of Black, Chinese, Hispanic, and White individuals. Participants were recruited in 2000–2002 from 6 communities across the U.S.: Forsyth County (Winston-Salem), NC; New York, NY; Baltimore, MD; St. Paul, MN; Chicago, IL; and Los Angeles, CA. The study team excluded females from this study. They included all men from MESA who completed the family history interview during Exam 2 (2002–2004) and had complete data for comorbidities and CVH, resulting in a sample size of 2,814 males (Figure 1). The 399 men who were excluded from this study for missing data or not participating in Exam 2 differed from those included in the analytic sample by race/ethnicity (excluded participants were a higher proportion Black and lower proportion White than those included in the sample, *p*<0.001), marital status (excluded sample had lower percentage married men, *p*=0.02), and depressive symptoms (excluded sample had a higher average depression score, *p*=0.01) (Appendix Table 1, available online). This study follows STROBE guideline reporting for cohort studies. The study was approved by the IRBs of each site, and all participants provided informed consent.

### Measures

Men were categorized as either fathers or nonfathers on the basis of information from the family history interview that was completed at Exam 2. In this interview, participants were asked to list any children's ages and medical conditions. Men who did not list any children were categorized as nonfathers. The authors calculated fathers’ age at birth of their oldest child (fatherhood onset) as the age of the participant at Exam 2 minus the current age of their oldest child at Exam 2. Because becoming a father at older ages is associated with improved health relative to becoming a father at a younger age, the authors used age categories that were used in previous articles and made age ≥35 years the reference group.^13^^,^^15^ One participant had a child between Exams 1 and 2 and was excluded from the analysis.

CVH was measured utilizing the American Heart Association's LE8 framework.^4^ The 8 metrics consist of 4 health behaviors (healthy diet, participation in physical activity, avoidance of nicotine, and healthy sleep) and 4 health factors (healthy weight, level of blood lipids, levels of blood glucose, and blood pressure). There were no data for sleep at Exam 1, and the LE8 framework is appropriate to use even without all 8 metrics,^26^ so this metric was excluded from our analysis. Participant information from Exam 1 was used to calculate a CVH score from 0 to 100 for each of the 7 metrics and average total CVH score of the 7 metrics; a higher score indicates a healthier metric. Data for diet,^27^^,^^28^ physical activity,^29^ and nicotine exposure^30^ were collected through questionnaires adopted from validated instruments. BMI (kg/m^2^) was calculated from measurements of weight and height. Total cholesterol and plasma glucose were measured from blood samples obtained after a 12-hour fast. Resting seated blood pressure was measured 3 times, and the mean of the last 2 measurements was used.

On the basis of previous literature,^3^^,^^6^^,^^19^^,^^20^^,^^22^^,^^31^, ^32^, ^33^ the covariates assessed were age, race/ethnicity, marital status, education, having a primary care clinician, total gross family income level, depressive symptoms, total serum testosterone, and alcohol consumption. All covariate data were collected from Exam 1. For 68 individuals with missing income data at Exam 1, Exam 2 data were used. Depression was defined as a Center for Epidemiologic Studies Depression (CESD) Scale score ≥16.

A detailed description of the follow-up methods is available at https://internal.mesa-nhlbi.org. Briefly, follow-up for medical events occurred for 18 years (2000–2018) at 9-to-12-month intervals through interviews and review of hospital records. Two physician members of the MESA morbidity and mortality review committee independently classified significant clinical events. If they disagreed, then the full committee made the final classification. CVD events were defined as definite or probable myocardial infarction, death due to coronary heart disease, resuscitated cardiac arrest, and coronary revascularization; fatal or nonfatal stroke (ischemic and hemorrhagic subtypes); definite or probable heart failure; peripheral arterial disease; and CVD death. CVD death was defined as death due to atherosclerotic heart disease, stroke, atherosclerotic disease other than coronary disease, and other CVD.

### Statistical Analysis

The associations between fatherhood status and demographic characteristics and CVH metrics were examined using 2-sided chi-square and *t*-test procedures. Multivariable linear regression and general linear models were used to assess the relationship of fatherhood status and onset with CVH. Multivariable Cox proportional hazard regression models were used to evaluate the association of fatherhood status and onset with time to incident CVD events, CVD death, and all-cause mortality. There was no evidence of violations of the proportionality assumption (*p*>0.05). The covariates were included sequentially with age only and then all of our covariates: marital status, education, having a primary care clinician, income, total serum testosterone, alcohol consumption, and CESD depression score. For the CVD and mortality analysis, the authors ran a model with all the covariates and then a second model with the covariates and total CVH score at Exam 1. Because there were no changes to significant results with CVH added, the authors present the fully adjusted model only. They assessed for effect modification by race/ethnicity and marital status on the association of fatherhood with each outcome. In addition, they ran their analyses as an overall sample and stratified by race/ethnicity. Analysis was performed using SAS, Version 9.4 (SAS Institute, Cary, NC).

## RESULTS

Among the 2,814 men, mean age at CVH assessment was 62.2 years, 24% self-identified as Black, 13% self-identified Chinese, 22% self-identified Hispanic, and 41% self-identified White (Table 1). Most men (82.4%) were fathers at the time of CVH assessment. Fathers were less likely to report White race (37.5% vs 56.7% for nonfathers, *p*<0.001) and a bachelor's degree or higher level of education (40.3% vs 53.6%, *p*<0.001) but were not more likely to report total gross family income ≥$50,000 (48.5% vs 44.1%, *p=*0.08) (Table 1) than nonfathers. Fathers reported lower levels of depressive symptoms at Exam 1 than nonfathers (7.2% vs 11.7% had CESD score ≥16, *p*<0.001). The mean age of fathers at the birth of their oldest child was 27.6 years (SD=6.3), and the average number of children among fathers was 2.9 (SD=1.7). There was a significant difference in the average age of fathers at the birth of their oldest child by race and ethnicity: 25.8 (SD=6.5) for Black men, 30.7 (SD=6.2) for Chinese men, 26.6 (SD=6.1) for Hispanic men, and 28.3 (SD=5.8) for White men (*p*<0.001) (Appendix Table 2, available online).

**Table 1:** Characteristics of MESA Sample at Baseline

Characteristic^a^	Fathers (*n*=2,320)	Nonfathers (*n*=494)	*p*-value^b^ (χ^2^)
Mean age total sample, years (SD)	62.5 (10.0)	60.7 (10.5)	<0.001
Race and ethnicity, *n* (%)			<0.001
Black	567 (24.4)	119 (24.1)	
Chinese	324 (14.0)	27 (5.5)	
Hispanic	559 (24.1)	68 (13.8)	
White	870 (37.5)	280 (56.7)	
Bachelor's degree or higher, *n* (%)	936 (40.3)	265 (53.6)	<0.001
Total family income >$50,000, *n* (%)	1,126 (48.5)	218 (44.1)	0.08
Married, *n* (%)	1,822 (78.5)	225 (45.6)	<0.001
Has primary care clinician, *n* (%)	2,127 (91.7)	456 (92.3)	0.65
Total serum testosterone level, *n* (SD)	14.8 (5.3)	15.1 (5.7)	0.33
CESD average score, *n* (SD)	6.0 (6.2)	7.3 (7.3)	<0.001
CESD score >16, *n* (%)	166 (7.2)	58 (11.7)	<0.001
Alcohol use, *n* (%)			0.21
Never	238 (10.3)	45 (9.1)	
Former	620 (26.7)	117 (23.7)	
Current	1,462 (63.0)	332 (67.2)	
Life's Essential 8 CVH score, *n* (0–100)	63.0 (13.9)	65.4 (14.6)	<0.001
Diet, *n* (SD)	38.4 (30.8)	41.1 (31.5)	0.07
Physical activity, *n* (SD)	74.8 (40.4)	74.8 (40.0)	0.97
Nicotine exposure, *n* (SD)	63.6 (33.3)	64.3 (33.1)	0.67
BMI, *n* (SD)	62.9 (27.5)	65.3 (30.3)	0.11
Blood lipids non-HDL cholesterol, *n* (SD)	63.2 (28.0)	64.6 (28.6)	0.31
Blood glucose, *n* (SD)	79.4 (25.2)	83.2 (23.5)	0.002
Blood pressure, *n* (SD)	59.0 (35.3)	64.6 (33.2)	0.001
Number of children, *n* (SD)	2.9 (1.7)	0 (0)	n/a
Age at oldest child's birth, *n* (SD)	27.6 (6.3)	n/a	n/a

a Characteristics recorded at Exam 1, except the number and age of children, which was recorded at Exam 2.

b Computed with chi-square across categories. ^c^Higher CVH score indicates a healthier metric. CESD, Center for Epidemiologic Studies Depression; CVH, cardiovascular health; HDL, high-density lipoprotein; MESA, Multi-Ethnic Study of Atherosclerosis; n/a, not available.

Fathers had worse overall CVH (adjusted mean score=63.2 vs 64.7, *p*=0.03) and more nicotine exposure (63.1 vs 66.6, *p*=0.04) than nonfathers. Otherwise, there was no difference in the other CVH metrics at Exam 1 between fathers and nonfathers (Table 2). After stratifying by race and ethnicity, the authors found that White fathers had worse total CVH than White nonfathers (65.8 vs 68.9, *p*=0.01), but there was no significant difference between fathers and nonfathers for Black, Chinese, or Hispanic participants (Appendix Table 3, available online).

**Table 2:** Association Between Fatherhood Status and Onset With CVH Score Using Multivariate Linear Regression

CVH metric at average age of 62.2 years^a^	Fatherhood status, LS mean^b^ (SE) (*n*=2,814) (score range=0–100)^c^		Age at the birth of the first child, LS mean^b^ (SE) (*n*=2,320) (score range=0–100)^c^				
	Fathers	Nonfathers	<20	20–24	25–29	30–34	>35 (ref)
Diet	38.3 (0.6)	41.3 (1.4)	36.0 (2.6)	36.1 (1.2)	38.9 (1.1)	40.4 (1.4)	39.9 (1.8)
Physical activity	75.3 (0.8)	72.6 (1.8)	72.5 (3.5)	73.9 (1.6)	77.1 (1.4)	74.4 (1.9)	72.5 (2.3)
Nicotine exposure	**63.1 (0.7)**	**66.6 (1.5)**	**54.3 (2.7)**	**60.6 (1.2)**	63.9 (1.1)	69.3 (1.5)	65.4 (1.9)
BMI	63.0 (0.6)	64.7 (1.3)	62.0 (2.3)	**60.4 (1.0)**	**62.7 (0.9)**	64.1 (1.3)	67.4 (1.6)
Blood lipids	63.0 (0.6)	65.1 (1.3)	64.9 (2.4)	62.4 (1.1)	**62.0 (1.0)**	63.9 (1.3)	66.0 (1.6)
Blood glucose	80.0 (0.5)	80.3 (1.1)	77.9 (2.0)	**77.1 (0.9)**	80.7 (0.8)	80.2 (1.1)	81.1 (1.4)
Blood pressure	59.5 (0.7)	62.2 (1.5)	61.8 (2.9)	56.9 (1.3)	58.9 (1.2)	60.5 (1.6)	60.4 (2.0)
Total CVH score at Exam 1	**63.2 (0.3)**	**64.7 (0.6)**	**61.2 (1.1)**	**61.0 (0.5)**	63.5 (0.5)	64.7 (0.7)	64.7 (0.8)

*Note:* Boldface indicates statistical significance (*p*<0.05) between fathers and nonfathers or between men who became fathers at age >35 years and those of other age categories.

a Individuals CVH metrics taken from Exam 1.

b LS mean. Covariates include age, race/ethnicity, marital status, education, having a primary care clinician, income, total serum testosterone, former and current alcohol consumption, and CESD depression score.

c Higher CVH score indicates a healthier metric. CESD, Center for Epidemiologic Studies Depression; CVH, cardiovascular health; LS, least square.

Fathers who were aged <20 years had more nicotine exposure (*p*<0.01) and worse total CVH score (*p*=0.01) than fathers who were aged >35 years at the birth of their first child; fathers who were aged 20–24 years had worse nicotine exposure (*p*=0.03), BMI (*p*<0.01), blood sugar scores (*p*=0.049), and total CVH (*p*<0.001); and fathers who were aged 25–29 years had worse BMI (*p*<0.01) and blood lipid scores (*p*=0.0) (Table 2). In the stratified analysis, Black fathers who were aged <20 years (*p*=0.03) or 20–24 years (*p*<0.01) and Hispanic fathers who were aged <20 years (*p*=0.01) or 20–24 years (*p*<0.01) at the birth of their oldest child had worse total CVH than fathers who were aged >35 years at the birth of their oldest child (Appendix Table 3, available online).

During a median 17.6 years of follow-up, there were 608 CVD events (28.0% cumulative incidence), 214 deaths due to CVD (10.0% cumulative incidence), and 854 deaths of any causes (34.4% cumulative incidence). In age-adjusted models, fathers who were aged <20 years (hazard ratio [HR]=1.82; 95% CI=1.27, 2.62) and 20–24 years (HR=1.39; 95% CI=1.07, 1.81) at the birth of their oldest child had a higher rate of all-cause mortality than fathers who were aged >35 years at the birth of their first child (Appendix Table 4, available online). In the fully adjusted models, there was no association between father's age at the birth of their first child and CVD events, CVD death, and all-cause mortality (Appendix Table 4, available online).

In age-adjusted and fully adjusted models, fathers and nonfathers did not differ in hazards for CVD and CVD death (Table 3). In age-adjusted models, fathers had a lower rate of all-cause mortality (HR=0.82; 95% CI=0.69, 0.98), but this relationship was not significant in our fully adjusted model (HR=0.86; 95% CI=0.72, 1.03) (Table 3). The relationship between fatherhood and all-cause mortality in an unadjusted survival curve differs for White men (Appendix Figure 1, available online), but this was not significant in adjusted models. Similarly, the authors observed evidence for statistical interaction between fatherhood and race/ethnicity on all-cause mortality before adjustment (interaction *p*=0.00) (Appendix Table 5, available online). After adjustment, this interaction was attenuated (*p*=0.36), and the authors did not observe evidence for an interaction between fatherhood and race/ethnicity on incident CVD or CVD death (all *p* for moderation >0.05). In the stratified age-adjusted models, Black fathers had a lower rate of all-cause mortality (HR=0.73; 95% CI=0.53, 1.00; *p=*0.049) than Black nonfathers, but this association was attenuated in fully adjusted models (HR=0.79; 95% CI=0.56, 1.09). There was no difference for CVD, CVD death, and all-cause mortality for Chinese, Hispanic, and White fathers compared with nonfathers (Table 3). The authors did not observe evidence for effect modification by marriage status in associations between fatherhood status and our outcomes (all *p* for moderation >0.05).

**Table 3:** CVD Event, CVD Death, and All-Cause Mortality Among Fathers and Nonfathers Stratified by Race/Ethnicity

Clinical outcome		Race and ethnicity			
	All	Black	Chinese	Hispanic	White
CVD events^a^					
Fathers, event rate^b^ (95% CI)	15.61 (14.32–17.03)	15.03 (12.57–17.98)	11.87 (9.17–15.35)	17.50 (14.79–20.72)	16.30 (14.18–18.73)
Nonfathers, event rate^b^ (95% CI)	13.58 (11.12–16.58)	19.11 (13.36–27.33)	9.85 (3.70–26.25)	10.16 (5.47–18.88)	12.65 (9.64–16.60)
Age-adjusted HR	1.04 (0.84–1.30)	0.79 (0.53–1.18)	1.30 (0.47–3.59)	1.73 (0.91–3.30)	0.99 (0.73–1.35)
Multivariable adjusted HR^c^ (95% CI)	1.10 (0.87–1.38)	0.87 (0.57–1.31)	1.63 (0.57–4.70)	1.71 (0.89–3.27)	1.08 (0.77–1.51)
CVD death^d^					
Fathers, event rate^b^ (95% CI)	4.76 (4.10–5.51)	5.62 (4.26–7.42)	3.85 (2.51–5.90)	4.96 (3.69–6.66)	4.43 (3.44–5.69)
Nonfathers, event rate^b^ (95% CI)	4.88 (3.55–6.70)	9.22 (5.65–15.04)	6.90 (2.23–21.41)	2.83 (0.91–8.78)	3.51 (2.15–5.72)
Age-adjusted HR	0.80 (0.56–1.14)	0.58 (0.33–1.02)	0.53 (0.16–1.79)	1.76 (0.55–5.68)	0.87 (0.50–1.51)
Multivariable adjusted HR^c^ (95% CI)	0.86 (0.60–1.25)	0.59 (0.33–1.06)	0.55 (0.15–2.00)	1.84 (0.57–5.97)	0.99 (0.54–1.81)
All-cause mortality					
Fathers, event rate^b^ (95% CI)	18.89 (17.54–20.34)	20.70 (17.91–23.91)	12.82 (10.14–16.20)	16.56 (14.09–19.46)	21.62 (19.30–24.22)
Nonfathers, event rate^b^ (95% CI)	19.89 (16.99–23.28)	27.07 (20.34–36.03)	20.71 (10.78–39.81)	22.65 (15.18–33.79)	16.43 (13.10–20.61)
Age-adjusted HR	**0.82** **(0.69–0.98)**	**0.73** **(0.53**–**0.999)**	0.63 (0.31–1.26)	0.73 (0.47–1.12)	0.92 (0.72–1.19)
Multivariable adjusted HR^c^ (95% CI)	0.86 (0.72–1.03)	0.79 (0.56–1.09)	0.68 (0.32–1.44)	0.73 (0.47–1.14)	0.95 (0.72–1.26)

*Note:* Boldface indicates statistical significance (*p*<0.05).

a CVD events defined as definite or probable myocardial infarction, death due to coronary heart disease, resuscitated cardiac arrest, and coronary revascularization; fatal or nonfatal stroke (ischemic and hemorrhagic subtypes); definite or probable HF; peripheral arterial disease; and CVD death.

b Incidence rate per 1,000 person years.

c HR of fathers compared with nonfathers in multivariable Cox proportional model. Covariates include age, marital status, education, having a primary care clinician, income, total serum testosterone, alcohol consumption, CESD depression score, and total CVH score at Exam 1.

d CVD death defined as death to atherosclerotic heart disease, stroke, atherosclerotic disease other than coronary disease, and other cardiovascular disease. CESD, Center for Epidemiologic Studies Depression; CVD, cardiovascular disease; CVH, cardiovascular health; HF, heart failure.

## DISCUSSION

This study advances understanding on the associations between fatherhood and CVH, incident CVD, and mortality in older age. The authors include longitudinal assessment of CVD among a multiethnic sample of men and use the updated LE8 CVH framework.^4^ They found that fathers have worse CVH and more nicotine exposure than nonfathers. They observed that Black men, Hispanic men, and men overall who became fathers at age <20 and 20–25 years have worse total CVH than those who became fathers at age >35 years. They also observed an interaction between fatherhood and race/ethnicity on all-cause mortality. In age-adjusted models overall and in the race/ethnicity subgroup of Black men, they observed that fathers had a lower rate of all-cause mortality rate than nonfathers, but these associations were attenuated in fully adjusted models. The results demonstrate that fatherhood is an important social determinant of health and that the relationship of fatherhood with CVH may differ by age at fatherhood and race/ethnicity.

This study's findings align with those of prior work that demonstrates an association between the age men become fathers and health outcomes.^13^, ^14^, ^15^^,^^34^ This study found that Black fathers, Hispanic fathers, and fathers overall who had their first child at ages <20 and 20–24 years had worse CVH later in life than fathers who were aged >35 years at the birth of the first child. Similarly, a British cohort study found that men who were aged <20 years at the birth of their first child had higher mid-life blood pressure and BMI, even after controlling for multiple social and behavior factors.^13^ Early-timed fatherhood is associated with lower financial stability,^35^ increased likelihood of multiple partnerships,^35^ and depressive symptoms,^36^ all of which may increase the risk of poor health behaviors and outcomes throughout the life course.

Moreover, in age-adjusted models, the authors found that men overall and Black men who transitioned to fatherhood at ages <20 and 20–24 years had a higher rate of all-cause mortality than those who entered fatherhood from age >35 years. Similar findings have been seen in other populations. Among a sample of Norwegian men, fathers who were aged <23 years at the birth of their first child had higher mortality than the other age groups.^15^ Among a Finnish cohort of siblings, men who entered fatherhood aged <24 years had higher mortality than their brothers who entered fatherhood at ages 25–26 years.^14^ This higher mortality may be due to poor health behaviors, differences in cognitive abilities and sociodemographic characteristics, or other mechanisms that are not completely understood.^23^^,^^34^ Therefore, future research along with clinical and public health interventions that focus on young fathers may have important health impacts for men across their life course.

This study found that White fathers and fathers overall had worse total CVH than nonfathers. These findings are similar to prior observations that fatherhood has been associated with CVD risk factors such as weight gain,^10^^,^^11^ a decrease in physical activity,^9^ and an increase in fat intake.^12^ The changes in health metrics suggest that the additional commitment of child care responsibilities and stress during the transition to fatherhood may make it difficult for men to maintain a healthy diet and exercise for weight management.^37^ A study following men as they transition to fatherhood found that men had the largest weight gain in the first 6 months after child birth.^38^ Thus, to further define these relationships, future studies should focus on the time men transition to fatherhood in longitudinal cohorts to measure the life course trajectory of CVH metrics.

This study's finding that fathers were more likely to use nicotine than nonfathers was in contrast to a prior evaluation from a sample of National Health and Nutrition Examination Survey men aged 20–59 years that reported that men who were living with children had lower odds of smoking than men living without children.^9^ The present study differs because it is comparing fathers with nonfathers rather than with those living with children, and the MESA population was significantly older (average age of 62.2 years) at the time of data collection. The transition to fatherhood has been associated with an increase in depression^36^^,^^39^ and anxiety,^40^ and these factors may contribute to nicotine use habits. Because smoking is the leading cause of preventable death in the U.S.,^41^ a better understanding of the relationship between fatherhood and tobacco use may help inform public health and clinical smoking-cessation programs.

This study found that fathers have lower age-adjusted all-cause mortality but no difference in CVD or CVD events. This study is not the first to observe an association between fatherhood and lower mortality^17^^,^^18^; however, this study found that fathers actually have worse CVH, suggesting that there may be factors outside of CVH that contribute to differences in all-cause mortality between fathers and nonfathers. First, fathers may have a more robust social support system, and social connectedness has been associated with mortality.^42^ Second, fathers may be more likely to have someone as their future caretaker (i.e., their children) and help them attend medical appointments and manage medications and treatments. Finally, MESA fathers had lower rates of depressive symptoms than nonfathers. Because depression is associated with mortality in men,^43^ it may be an important mediator in the relationship between fatherhood and mortality. This study found that the relationship between mortality and fatherhood was attenuated with covariates; therefore, future studies are needed to evaluate the social factors, such as social support, that may mediate the relationship between fatherhood and mortality.

This study's finding of an interaction between race/ethnicity and fatherhood on all-cause mortality is a new development. The stratified age-adjusted analysis found that Black fathers had lower rates of all-cause mortality than nonfathers. Fatherhood has been associated with positive behavioral changes among Black men.^44^ Black men suffer from one of the lowest life expectancies of any demographic group in the U.S.^1^; future studies that focus on understanding the influence of fatherhood on health outcomes can illuminate opportunities for health promotion for Black men with and without children.

### Limitations

This study has several limitations. First, the average age of participants in this sample at the time of baseline health assessment was 62 years, so this study cannot measure the relationship between the transition to fatherhood and health; rather, the focus is on being a father and the association with CVH, CVD, and mortality. Second, MESA enrolled individuals who were free of CVD at baseline, and there is potential for selection bias if there is a difference between early-onset CVD among fathers and nonfathers. Third, it is possible that individuals became fathers after Exam 2, which would cause them to be miscategorized; however, in the U.S., only 3% of men become fathers over the age of 40 years.^6^ Fourth, data on male fertility were not available. Male infertility is associated with poor health outcomes,^21^ and this may be an important unmeasured confounder in this analysis. Fifth, the authors did not have information on whom men live with or time spent caring for children. Therefore, they cannot assess the relationship of CVH and mortality with caring for, living with, or being engaged with children. Sixth, variables measured at Exam 1 do not necessarily represent the participants’ characteristics at the time men became fathers, which could influence the relationship between fatherhood and health over the life course; for example, men might have been unmarried when they had children but recently married. Finally, there may be limits to this study's generalizability if the trends of fatherhood have evolved over the past several decades^45^ because most of the participants in the MESA sample became fathers in the 1970–1980s.

## CONCLUSIONS

In this multiethnic longitudinal sample, fatherhood status and age at entry to fatherhood were associated with CVH and age-adjusted mortality outcomes. This study highlights that men who transition to fatherhood at younger ages, especially Black and Hispanic men, may be at higher risk for poor health outcomes and may benefit from focused clinical and public health attention. The interaction between race/ethnicity and fatherhood on mortality underscores the complex influence of fatherhood on the health of men and suggests possible additional benefits of fatherhood for Black men. The life expectancy of men has stagnated over the past decade and actually decreased during the coronavirus disease 2019 (COVID-19) pandemic.^1^ Because most men in the U.S. are fathers,^6^ identifying some of the explanations for the associations between health, disease, and fatherhood could have important health implications for men, especially for men of color.

## CRediT authorship contribution statement

**John James F. Parker:** Conceptualization, Methodology, Investigation, Writing – original draft. **Craig F. Garfield:** Conceptualization, Methodology, Writing – review & editing. **Clarissa D. Simon:** Conceptualization, Methodology, Writing – review & editing. **Laura A. Colangelo:** Conceptualization, Methodology, Data curation, Formal analysis, Writing – review & editing. **Michael P. Bancks:** Conceptualization, Methodology, Writing – review & editing. **Norrina B. Allen:** Conceptualization, Methodology, Investigation, Supervision, Writing – review & editing.

## Appendix Supplementary materials

Image, application 1

## Footnotes

Supplementary material associated with this article can be found in the online version at doi:10.1016/j.focus.2024.100231.
