# Supplementary material for: Fatherhood and Cardiovascular Health, Disease, and Mortality: Associations From the Multi-Ethnic Study of Atherosclerosis
Source: AJPM Focus. 2024 May 6;3(4):100231. doi: 10.1016/j.focus.2024.100231 (PMC11179579; doi:10.1016/j.focus.2024.100231)
Supplement: Supplementary file 1 [file mmc1.docx]

**Appendix**

**Appendix Table 1.** Demographic characteristics among study sample and excluded participants

| **Characteristic** | **Study Sample (n=2814)** | **Excluded Participants (n=399)** | **P-value^a^** |
| --- | --- | --- | --- |
| Mean age, y (SD) | 62.2 (10.1) | 62.2 (10.9) | 0.99 |
| Race and Ethnicity, % |  |  |  |
| White | 1150 (40.9) | 110 (27.6) | <.0001 |
| Chinese | 351 (12.5) | 39 (9.8) |  |
| Black | 686 (24.4) | 156 (39.1) |  |
| Hispanic | 627 (22.3) | 94 (23.6) |  |
| Married, % | 2047 (72.7) | 261 (67.1) | 0.02 |
| CESD average score (SD) | 6.3 (6.4) | 7.3 (7.5) | 0.01 |

a Computed with chi-square across categories

**Appendix Table 2.** Characteristics of MESA Sample at Baseline by race/ethnicity

| **Characteristic^a^** | **Fathers (n=2320)** | **Nonfathers (n=494)** | **P-value^b^ (χ^2^)** |
| --- | --- | --- | --- |
| Mean age total sample, y (SD) | 62.5 (10.0) | 60.7 (10.5) | <0.001 |
| Mean age Black men, y (SD) | 61.9 (9.8) | 62.5 (10.3) | 0.56 |
| Mean age Chinese men, y (SD) | 62.0 (10.2) | 64.1 (9.2) | 0.30 |
| Mean age Hispanic men, y (SD) | 61.3 (10.1) | 61.5 (11.4) | 0.90 |
| Mean age White men, y (SD) | 63.8 (9.8) | 59.4 (10.3) | <0.001 |
| Bachelor’s degree or higher, n (%) | 936 (40.3) | 265 (53.6) | <0.001 |
| Black, n (%) | 195 (34.4) | 45 (37.8) | 0.47 |
| Chinese, n (%) | 170 (52.5) | 16 (59.3) | 0.50 |
| Hispanic, n (%) | 67 (12.0) | 14 (20.6) | 0.045 |
| White, n (%) | 504 (57.9) | 190 (67.9) | <0.01 |
| Total family income >$50,000 (%) | 1126 (48.5) | 218 (44.1) | 0.08 |
| Black, n (%) | 277 (48.9) | 42 (35.3) | <0.01 |
| Chinese, n (%) | 113 (34.9) | 7 (25.9) | 0.35 |
| Hispanic, n (%) | 134 (24.0) | 14 (20.6) | 0.53 |
| White, n (%) | 602 (69.2) | 155 (55.4) | <0.001 |
| Married, n (%) | 1822 (78.5) | 225 (45.6) | <0.001 |
| Black, n (%) | 361 (63.7) | 49 (41.2) | <0.001 |
| Chinese, n (%) | 301 (92.9) | 17 (63.0) | <0.001 |
| Hispanic, n (%) | 418 (74.8) | 34 (50.0) | <0.001 |
| White, n (%) | 742 (85.3) | 125 (44.6) | <0.001 |
| Has primary care clinician, n (%) | 2127 (91.7) | 456 (92.3) | 0.65 |
| Black, n (%) | 510 (90.0) | 109 (91.6) | 0.58 |
| Chinese, n (%) | 306 (94.4) | 25 (92.6) | 0.69 |
| Hispanic, n (%) | 469 (83.9) | 63 (92.7) | 0.06 |
| White, n (%) | 842 (96.8) | 259 (92.5) | <0.01 |
| Total serum testosterone level (SD) | 14.8 (5.3) | 15.1 (5.7) | 0.33 |
| Black, level (SD) | 14.9 (6.0) | 15.3 (7.2) | 0.42 |
| Chinese, level (SD) | 14.5 (4.8) | 14.1 (4.7) | 0.68 |
| Hispanic, level (SD) | 14.7 (4.9) | 15.3 (4.5) | 0.31 |
| White, level (SD) | 15.0 (5.4) | 15.0 (5.3) | 0.94 |
| CESD average score (SD) | 6.0 (6.2) | 7.3 (7.3) | <0.001 |
| Black, score (SD) | 5.9 (6.0) | 6.6 (6.7) | 0.21 |
| Chinese, score (SD) | 5.1 (5.3) | 4.7 (4.4) | 0.66 |
| Hispanic, score (SD) | 7.7 (7.6) | 9.0 (8.8) | 0.08 |
| White, score (SD) | 5.4 (5.5) | 7.5 (7.3) | <0.001 |
| Current alcohol use, n (%) |  |  |  |
| Black, n (%) | 316 (55.7) | 68 (57.1) | 0.84 |
| Chinese, n (%) | 149 (46.0) | 8 (29.6) | 0.20 |
| Hispanic, n (%) | 333 (59.6) | 42 (61.8) | 0.14 |
| White, n (%) | 664 (76.7) | 214 (76.4) | 0.77 |
| Age at oldest child’s birth (SD) | 27.6 (6.3) | n/a |  |
| Black, average age (SD) | 25.8 (6.5) | n/a | <0.001^d^ |
| Chinese, average age (SD) | 30.7 (6.2) | n/a | <0.001^d^ |
| Hispanic, average age (SD) | 26.6 (6.1) | n/a | <0.001^d^ |
| White, average age (SD) | 28.3 (5.8) | n/a | ref |

^a^ Characteristics recorded at exam 1, except number and age of children which was recorded at exam 2

^b^ Computed with chi-squared across categories

^c^ Higher CVH score indicates a healthier metric

^d^ Pairwise comparison of age at fatherhood onset for Black, Chinese and Hispanic father compared with white fathers

**Appendix Table 3.** Association between fatherhood status and onset with total CVH score using multivariate linear regression stratified by race and ethnicity

| **Total mean CVH score^a^** | **Fatherhood Status, LS Mean^b^ (SE) (n=2814) (Score range 0-100)^c^** | | **Age at the birth of the first child, LS Mean^b^ (SE) (n=2320) (Score range 0-100)^c^** | | | | |
| --- | --- | --- | --- | --- | --- | --- | --- |
|  | Fathers | Nonfathers | <20 | 20-24 | 25-29 | 30-34 | >35 (ref) |
| Black | 60.0 (0.6) | 61.0 (1.3) | **59.0 (1.6)** | **58.8 (1.0)** | **59.3 (1.1)** | 62.0 (1.6) | 64.7 (2.0) |
| Chinese | 65.9 (0.7) | 64.4 (2.6) | 61.3 (6.0) | 62.6 (2.3) | 66.7 (1.2) | 65.8 (1.3) | 66.4 (1.7) |
| Hispanic | 60.1 (0.6) | 60.0 (1.6) | **56.2 (2.2)** | **58.2 (0.9)** | 61.3 (1.0) | 61.2 (1.5) | 63.6 (1.8) |
| White | **65.9 (0.4)** | **68.6 (0.8)** | 64.2 (2.9) | 63.4 (0.8) | 66.1 (0.7) | 68.5 (0.9) | 66.2 (1.2) |
| All races | **63.2 (0.3)** | **64.7 (0.6)** | **61.1 (1.2)** | **61.0 (0.5)** | 63.5 (0.5) | 64.7 (0.6) | 64.7 (0.8) |

^a^ Individuals CVH metrics taken from Exam 1

^b^ Least Squares Mean. Covariates include age, race/ethnicity, marital status, education, having a primary care clinician, income, number of children, total serum testosterone, former and current alcohol consumption & CESD depression score.

^c^Higher CVH score indicates a healthier metric

Boldface indicates statistical significance (*p*<0.05) between fathers and Nonfathers or men who became fathers >35 compared with other age categories.

**Appendix Table 4.** CVD event, CVD death and all-cause mortality by father’s age at the birth of the oldest child

|  | **Age at the birth of the first child (n=1944)** | | | | |
| --- | --- | --- | --- | --- | --- |
| **Clinical Outcome** | **<20** | **20-24** | **25-29** | **30-34** | **>35** |
| **CVD events^a^** |  |  |  |  |  |
| Event rate^b^ (95%CI) | 11.98  (7.96, 18.03) | 17.67  (15.14, 20.62) | 15.27  (13.15, 17.73) | 14.02  (11.41, 17.24) | 16.18  (12.71, 20.60) |
| Age-adjusted Hazard Ratio (95%CI) | 0.88 (0.55, 1.42) | 1.21 (0.91, 1.61) | 0.93 (0.70, 1.24) | 0.91 (0.66, 1.26) | ref |
| Adjusted Hazard Ratio^c^ (95%CI) | 0.73 (0.44, 1.22) | 1.01 (0.74, 1.39) | 0.88 (0.65, 1.17) | 0.92 (0.67, 1.27) | ref |
| **CVD death^d^** |  |  |  |  |  |
| Event rate^b^ (95%CI) | 5.56  (3.16, 9,78) | 5.25  (4.03, 6.84) | 4.82  (3.75, 6.20) | 3.77  (2.59, 5.50) | 4.58  (2.99, 7.03) |
| Age-adjusted Hazard Ratio (95%CI) | 1.78 (0.87, 3.62) | 1.46 (0.88, 2.43) | 1.07 (0.65, 1.76) | 0.89 (0.51, 1.58) | ref |
| Adjusted Hazard Ratio^c^ (95%CI) | 1.26 (0.59, 2.71) | 1.14 (0.66, 1.96) | 0.95 (0.57, 1.6) | 0.87 (0.49, 1.55) | ref |
| **All-cause mortality** |  |  |  |  |  |
| Event rate^b^ (95%CI) | 21.76  (16.35, 28.96) | 18.91  (16.45, 21.73) | 19.54  (17.25, 22.13) | 18.03  (15.17, 21.42) | 17.03  (13.64, 21.26) |
| Age-adjusted Hazard Ratio (95%CI) | **1.82 (1.27, 2.62)** | **1.39 (1.07, 1.81)** | 1.17 (0.9, 1.51) | 1.15 (0.87, 1.52) | ref |
| Adjusted Hazard Ratio^c^ (95%CI) | 1.39 (0.94, 2.05) | 1.12 (0.85, 1.49) | 1.04 (0.8, 1.36) | 1.11 (0.84, 1.48) | ref |

^a^CVD events defined as definite or probable myocardial infarction, death due to coronary heart disease, resuscitated cardiac arrest, and coronary revascularization, fatal or nonfatal stroke (ischemic and hemorrhagic subtypes), definite or probable heart failure (HF), peripheral arterial disease, and CVD death

^b^Incidence rate per 1000-person years

^c^Hazard Ratio of fathers compared to Nonfathers in multivariable Cox proportional model. Covariates include age, marital status, education, having a primary care clinician, income, total serum testosterone, alcohol consumption & CESD depression score >16, and total CVH score at exam 1

^d^CVD death defined as death to atherosclerotic heart disease, stroke, atherosclerotic disease other than coronary disease, and other cardiovascular disease

Boldface indicates statistical significance (*p*<0.05)

**Appendix Table 5.** Interaction analysis of Fatherhood and Race and Ethnicity on CVD event, CVD death and all-cause mortality

|  | Race/ethnicity and fatherhood status interaction p-value | Black vs White interaction p-value | White vs Chinese interaction p-value | White vs Hispanic interaction p-value |
| --- | --- | --- | --- | --- |
| CVD, unadjusted | 0.13 | **0.05** | 0.89 | 0.42 |
| CVD, adjusted | 0.20 | 0.34 | 0.53 | 0.14 |
| CVD death, unadjusted | 0.13 | **0.06** | 0.20 | 0.65 |
| CVD death, adjusted | 0.33 | 0.24 | 0.43 | 0.38 |
| All-cause mortality, unadjusted | **0.007** | **0.005** | **0.03** | **0.02** |
| All-cause mortality, adjusted | 0.36 | 0.19 | 0.22 | 0.22 |

Boldface indicates statistical significance (*p*<0.05)

**Appendix Table 6.** Association between age at fatherhood onset with CVH score using multivariate linear regression and adding number of children as a covariate

| **CVH metric^a^** | **Age at the birth of the first child, LS Mean^c^ (SE) (n=2320) (Score range 0-100)^d^** | | | | |
| --- | --- | --- | --- | --- | --- |
|  | <20 | 20-24 | 25-29 | 30-34 | >35 (ref) |
| Diet | 36.4 (2.6) | 36.2 (1.2) | 38.9 (1.1) | 40.3 (1.4) | 39.6 (1.8) |
| Physical Activity | 72.9 (3.5) | 74.0 (1.6) | 77.1 (1.4) | 74.3 (1.9) | 72.2 (2.4) |
| Nicotine Exposure | **53.1 (2.8)** | **60.1 (1.3)** | 63.9 (1.1) | 69.7 (1.5) | 66.4 (1.9) |
| Body mass index | 61.8 (2.3) | **60.4 (1.1)** | **62.7 (0.9)** | 64.1 (1.3) | 67.5 (1.6) |
| Blood lipids | 64.9 (2.4) | 62.4 (1.1) | **62.0 (1.0)** | 63.9 (1.3) | 66.1 (1.7) |
| Blood Glucose | 77.3 (2.1) | **77.2 (0.9)** | 80.7 (0.8) | 80.2 (1.1) | 80.7 (1.4) |
| Blood Pressure | 61.6 (2.9) | 56.8 (1.3) | 58.9 (1.2) | 60.5 (1.6) | 60.6 (2.0) |
| Total CVH score at exam 1 | **61.1 (1.2)** | **61.0 (0.5)** | 63.5 (0.5) | 64.7 (0.6) | 64.7 (0.8) |

^a^ Individuals CVH metrics taken from Exam 1

^b^ Least Squares Mean. Covariates include age, race/ethnicity, marital status, education, having a primary care clinician, income, total serum testosterone, former and current alcohol consumption & CESD depression score.

^c^ Least Squares Mean. Covariates include age, race/ethnicity, marital status, education, having a primary care clinician, income, **number of children**, total serum testosterone, former and current alcohol consumption & CESD depression score.

^d^Higher CVH score indicates a healthier metric

Boldface indicates statistical significance (*p*<0.05) between fathers and Nonfathers or men who became fathers >35 compared with other age categories.

**Appendix Figure 1.** Survival Curve, all-cause mortality among fathers and Nonfathers stratified by race/ethnicity


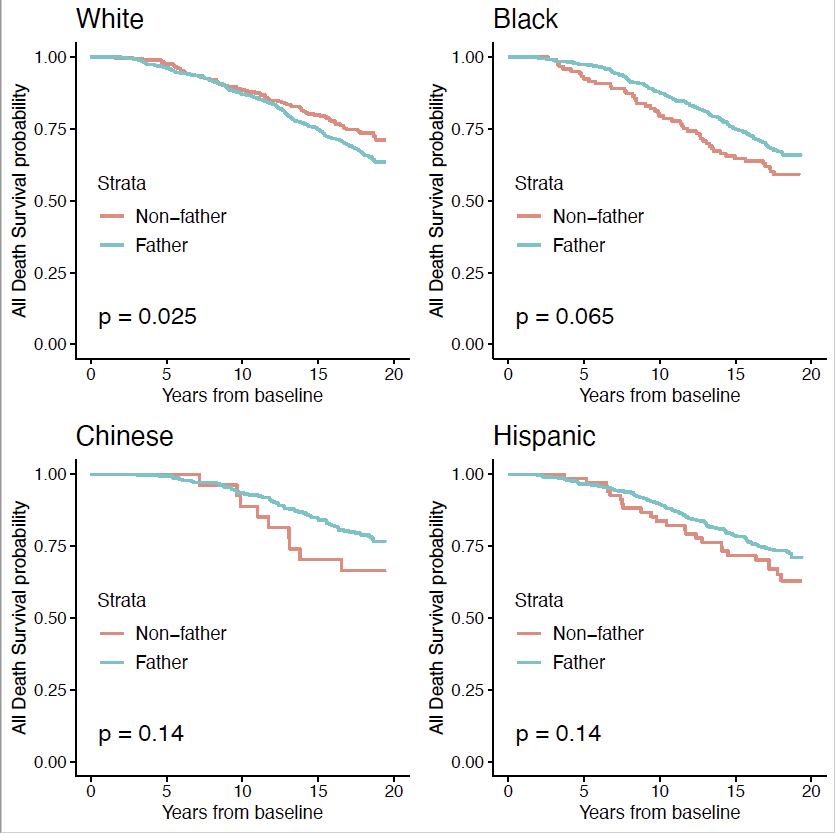


^d^CVD death defined as death to atherosclerotic heart disease, stroke, atherosclerotic disease other than coronary disease, and other cardiovascular disease

Boldface indicates statistical significance (*p*<0.05)
